# Nanobeam precession-assisted 3D electron diffraction reveals a new polymorph of hen egg-white lysozyme

**DOI:** 10.1107/S2052252518017657

**Published:** 2019-01-15

**Authors:** Arianna Lanza, Eleonora Margheritis, Enrico Mugnaioli, Valentina Cappello, Gianpiero Garau, Mauro Gemmi

**Affiliations:** a Center for Nanotechnology Innovation@NEST, Istituto Italiano di Tecnologia, Piazza San Silvestro 12, 56127 Pisa, Italy

**Keywords:** macromolecular crystallography, electron crystallography, nanobeam pressure-assisted electron diffraction, monoclinic lysozyme, radiation damage

## Abstract

Recent advances in 3D electron diffraction have allowed the structure determination of several model proteins from submicrometric crystals, the unit-cell parameters and structures of which could be immediately validated by known models previously obtained by X-ray crystallography. Here, the first new protein structure determined by 3D electron diffraction data is presented: a previously unobserved polymorph of hen egg-white lysozyme. This form, with unit-cell parameters *a* = 31.9, *b* = 54.4, *c* = 71.8 Å, β = 98.8°, grows as needle-shaped submicrometric crystals simply by vapor diffusion starting from previously reported crystallization conditions. Remarkably, the data were collected using a low-dose stepwise experimental setup consisting of a precession-assisted nanobeam of ∼150 nm, which has never previously been applied for solving protein structures. The crystal structure was additionally validated using X-ray synchrotron-radiation sources by both powder diffraction and single-crystal micro-diffraction. 3D electron diffraction can be used for the structural characterization of submicrometric macromolecular crystals and is able to identify novel protein polymorphs that are hardly visible in conventional X-ray diffraction experiments. Additionally, the analysis, which was performed on both nanocrystals and microcrystals from the same crystallization drop, suggests that an integrated view from 3D electron diffraction and X-ray microfocus diffraction can be applied to obtain insights into the molecular dynamics during protein crystal growth.

## Abstract 2

Stepwise nanobeam precession-assisted electron diffraction allows the structure determination of macromolecular nanocrystals, while limiting radiation damage. A new monoclinic polymorph of lysozyme was revealed and analyzed.

## 1. Introduction   

In order to address many challenging scientific issues concerning structural biology, several X-ray microfocus beamlines worldwide are fully dedicated to the analysis of 3D protein crystals smaller than a few tens of micrometres (Smith *et al.*, 2012 ▸). The relevance of nanocrystallography is driving some beamlines to achieve beam sizes below 1 µm in order to investigate even smaller protein crystals (Moukhamet­zianov *et al.*, 2008 ▸; Owen *et al.*, 2016 ▸). However, access for the scientific community to X-ray microfocus beamlines or to unconventional approaches such as X-ray free-electron lasers (XFELs; McNeil & Thompson, 2010 ▸) is strongly limited (Grimes *et al.*, 2018 ▸), and therefore there is growing interest in the development of alternative approaches. In this regard, electron-microscopy methods appear to be particularly promising. Cryo-EM imaging has rapidly become a widespread technique that is able to skip the crystallization step by directly imaging macromolecular complexes or potentially even single molecules of sufficient size (Amunts *et al.*, 2014 ▸; Kühlbrandt, 2014 ▸; Cheng, 2015 ▸; Nogales & Scheres, 2015 ▸; Fernandez-Leiro & Scheres, 2016 ▸; Quentin & Raunser, 2018 ▸).

Electron diffraction (ED) is another interesting option for the study of submicrometric protein crystals. It can be implemented in standard, relatively affordable, transmission electron microscopes (TEM) after simple hardware upgrades that include a fast and highly sensitive camera and possibly a beam-precession device; it is applicable to a wide range of compounds of different molecular weights and is also capable of quasi-atomic resolution.

ED has been applied to biological macromolecules since 1975, immediately providing highly relevant results (Unwin & Henderson, 1975 ▸; Henderson & Unwin, 1975 ▸). However, this initial approach was based on the acquisition of patterns oriented along main crystallographic axes and was limited to a few exceptionally stable systems capable of forming 2D crystals (for example some membrane proteins). The extension of this method to 3D crystals was mostly hampered by the strong dynamic effects arising in oriented low-index diffraction zones, which spoil the linear relation between the diffracted intensities and the square modulus of the structure factors (Gemmi *et al.*, 2003 ▸).

Recently, it has been demonstrated that if ED data are collected ‘off-zone’ by tilting the crystal, either in steps or continuously, around the goniometer axis, then the dynamic scattering can be significantly reduced and the ED intensities can be used as kinematic to solve the crystal structure *ab initio* (Kolb *et al.*, 2007 ▸, 2010 ▸; Mugnaioli *et al.*, 2009 ▸; Zhang *et al.*, 2010 ▸; Gemmi *et al.*, 2015 ▸). The successful application of the method to inorganic crystals, where the presence of heavy elements enhances the dynamic scattering (Jiang *et al.*, 2011 ▸; Guo *et al.*, 2015 ▸; Palatinus *et al.*, 2017 ▸; Rozhdestvenskaya *et al.*, 2017 ▸; Simancas *et al.*, 2016 ▸), guarantees that ED is suitable for the investigation of any type of crystal structure, provided that the crystal is not damaged by the conditions inside a TEM: a 10^−5^ Pa vacuum and a 100–300 kV electron beam.

Starting from 2013, ED has successfully been applied to 3D protein crystals that are too thin for X-ray diffraction or, at least, that are smaller than ∼500 nm (Nederlof *et al.*, 2013 ▸; Shi *et al.*, 2013 ▸; Nannenga *et al.*, 2014 ▸; Yonekura *et al.*, 2015 ▸; Nannenga & Gonen, 2016 ▸; Clabbers *et al.*, 2017 ▸; Xu *et al.*, 2018 ▸). In certain cases, ED data even allowed the *ab initio* localization and refinement of H atoms in inorganic and organic crystal structures of various complexities (Palatinus *et al.*, 2017 ▸; Rodriguez *et al.*, 2015 ▸). Because the same technique, with slight experimental modifications, has been given several names by different authors (ADT, RED, EDT, PEDT, FEDT and MicroED), we prefer in this paper to simply refer to it as 3D electron diffraction (3D ED). For comprehensive reviews of different data-collection strategies, we refer to Gorelik *et al.* (2011 ▸), Gemmi & Oleynikov (2013 ▸), Nannenga *et al.* (2014 ▸), Gemmi *et al.* (2015 ▸), Hattne *et al.* (2015 ▸), Mugnaioli & Kolb (2015 ▸) and Shi *et al.* (2016 ▸).

During 3D ED experiments, electrons interact strongly with the ordered assembly of macromolecules, making it possible to examine submicrometric crystals (Glaeser & Ceska, 1989 ▸; Henderson, 1995 ▸). Exploiting the quasi-kinematic character of ED intensities in 3D experiments, ED data were efficiently used for the structure determination of protein molecules by molecular replacement (Hattne *et al.*, 2015 ▸). Phase determination by *ab initio* techniques and multiple isomorphous replacements has also been hypothesized in ED (Dorset, 1995 ▸; Burmester & Schröder, 1997 ▸; Liu *et al.*, 2017 ▸). An interesting feature of ED is that the pattern formed by elastically scattered electrons is directly related to the distribution of the Coulomb potential, which contains information on the charged states of protein metal centers and residues (Yonekura *et al.*, 2015 ▸).

Hen egg-white lysozyme (HEWL) is one of the protein structures that have been most investigated by 3D ED to date. HEWL has an ability to crystallize easily in different lattices and space groups, and this characteristic has been greatly exploited in X-ray studies devoted to surveying different protein crystallization techniques (Alderton & Fevold, 1946 ▸; Strynadka & James, 1996 ▸; Chayen *et al.*, 1993 ▸; Alberstein & Tezcan, 2018 ▸).

Overall, more than 700 structures of HEWL have been solved, mostly using the standard single-crystal X-ray method, and deposited in the PDB. At present, among the deposited HEWL structures, 540 belong to the most common tetragonal form *P*4_3_2_1_2 (Chayen & Saridakis, 2001 ▸) with one molecule in the asymmetric unit, and 52 belong to the monoclinic space group *P*2_1_ with one or two molecules in the asymmetric unit (Harata, 1994 ▸). Another ten of these deposited structures belong to the orthorhombic space group *P*2_1_2_1_2, while a few others belong to the triclinic space group *P*1 (Ramanadham *et al.*, 1990 ▸) or the hexagonal space group *P*6_1_22 (Brinkmann *et al.*, 2006 ▸).

Two different polymorphs of HEWL have been determined by 3D ED to date. ED data collected in discrete rotation steps were used to solve the structure of the most common tetragonal HEWL polymorph at 2.9 Å resolution (space group *P*4_3_2_1_2, unit-cell parameters *a* ≃ 77, *c* ≃ 37 Å), merging data from three microcrystals (Shi *et al.*, 2013 ▸). Later, the same polymorph was also determined using continuous-rotation ED data (Nannenga *et al.*, 2014 ▸; Shi *et al.*, 2016 ▸). Additionally, a rarer HEWL orthorhombic polymorph with space group *P*2_1_2_1_2 and unit-cell parameters *a* ≃ 104, *b* ≃ 68, *c* ≃ 32 Å was determined independently by two different groups using continuous-rotation ED data collection (Clabbers *et al.*, 2017 ▸; Xu *et al.*, 2018 ▸). Both of these polymorphic forms had previously been characterized by X-ray diffraction analysis (Blake *et al.*, 1965 ▸; Sharma *et al.*, 2016 ▸).

Here, we report the structure determination of a novel, previously unobserved, monoclinic polymorph of HEWL, which was achieved using a 3D ED experimental setup that was originally developed for nanosized small-molecule crystals and designed to minimize the electron dose on the sample. Data were recorded in discrete steps using a nanodiffraction configuration that has never previously been applied for macromolecules. Such a configuration allows the illumination of only a fraction of the crystal at a time, avoiding damage to the non­diffracting area.

Such diffraction experiments were performed using a TEM operating at the relatively low voltage of only 120 kV instead of the commonly used 200 or 300 kV instruments. This is remarkable for two reasons. It demonstrates that dynamic effects are not a critical issue for the 3D ED data-collection geometry, since 3D ED data are also reliable for structure solution at voltages where the dynamic scattering is stronger. Secondly, the technique is portable to less expensive instruments such as low-voltage TEMs.

**Figure 1:**
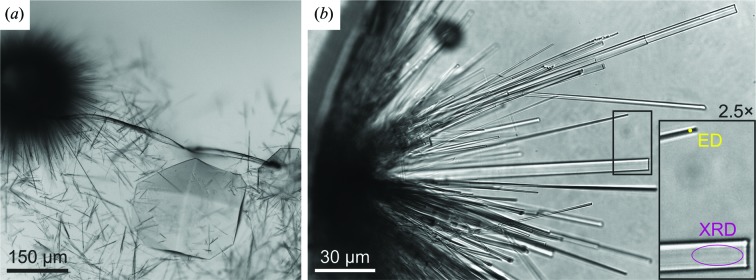
HEWL crystals. (*a*) Crystallization drop containing the needle-shaped microcrystals (dark gray) belonging to the novel monoclinic polymorph investigated by 3D ED and reported in this study. The drop also contains one common tetragonal crystal (light gray). (*b*) An aggregate of acicular crystals of different sizes grown during several weeks. The inset shows two crystals suitable for X-ray diffraction and ED, respectively, at a higher magnification and compared with the beam sizes used in this study. The purple ellipse represents the 10 × 4 µm beam of the microfocus X-ray diffraction beamline ID23-2 at the ESRF; the yellow dot is four times larger than the diameter of the electron nanobeam produced by the Zeiss Libra 120 TEM used in this study.

In order to validate the novel HEWL crystal form, we eventually collected synchrotron X-ray powder diffraction from an aggregate of submicrometric crystals (Fig. 1 ▸ *a*) and a microfocus single-crystal X-ray diffraction data set taken from a microcrystal grown after several weeks in the same crystallization drop (Fig. 1 ▸ *b*). Remarkably, such a crystal was just at the limit of the useful size for withstanding the X-ray radiation dose (Sliz *et al.*, 2003 ▸).

Besides allowing the solution of unknown structures of biological macromolecules from very small crystals, ED may be used also to discover novel and possibly metastable polymorphs and even to follow the structural evolution at different stages of crystallogenesis.

## 2. Methods   

### 2.1. Protein crystallization   

Lyophilized lysozyme from chicken egg white (Sigma–Aldrich) was dissolved in filtered water to a final concentration of 20 mg ml^−1^. Crystals were grown using the hanging-drop vapor-diffusion method from an unbuffered 1:1 mixture of sample solution and reservoir solution consisting of 1.5 *M* NaCl, starting from the reported crystallization conditions (Berthou *et al.*, 1983 ▸; Vaney *et al.*, 2001 ▸). After a few hours of incubation at room temperature, aggregates of needle-shaped crystals appeared, together with tetragonal HEWL crystals (Fig. 1 ▸ *a*). Hedgehog-like aggregates of micro-acicular crystals were disassembled by gently pipetting up and down in a clean drop of the same reservoir solution as used for crystallization.

### 2.2. Electron crystallography   

#### 2.2.1. Sample preparation   

Crystals were deposited on copper grids coated with Quantifoil R 2/1 holey films (EMS) with regular circular holes of 2 µm in diameter. Before deposition, the grids were treated with an oxygen plasma cleaner for 30 s at 20 W and they were used within 1 h to maintain a stable hydrophilicity. The grids were incubated horizontally with 5 µl of the suspension containing the HEWL crystals for 5 min at room temperature and then mounted on a Leica EM GP cryo-plunging apparatus. The environmental chamber was set to 4°C and 60% relative humidity. The grids were blotted for 1.5 s and plunge-frozen into liquid ethane at a temperature of 98 ± 3 K. Plunged grids collected in grid boxes were finally stored in liquid nitrogen and quickly moved to the transfer station for loading into the cryo-holder. Typical sample images are shown in Supplementary Fig. S1.

#### 2.2.2. Data collection   

Electron diffraction experiments were performed on a Zeiss Libra 120 TEM operating at 120 kV and equipped with an LaB_6_ thermionic source and an in-column omega filter. The data were obtained in stepwise mode, as reported by Kolb *et al.* (2007 ▸) and Mugnaioli *et al.* (2009 ▸) for small-molecule compounds. Diffraction patterns were collected in Köhler parallel illumination with a beam of ∼150 nm obtained using a 5 µm condenser aperture. The electron beam was precessed around a semi-angle of 0.85° by a NanoMEGAS DigiSTAR P1000 device (Vincent & Midgley, 1994 ▸; Midgley & Eggeman, 2015 ▸). All of the patterns were energy-filtered on the zero-loss peak with a slit width of ∼20 eV. Sequential diffraction patterns were recorded in tilt steps of 1° inside a full tilt range of 91°. After each tilt step, the crystal position was tracked in dark-field STEM imaging mode, keeping the same parallel beam as used for diffraction. Such a defocused beam produced blurred images, but these were sufficient to detect the crystal areas of interest and re-center them into the beam. This approach allowed us to only illuminate the crystal during acquisition of the diffraction pattern (∼2 s) and the STEM image (fractions of a second). Diffraction data were recorded using an ASI Timepix detector (Llopart *et al.*, 2007 ▸) that was able to record the arrival of single electrons and deliver virtually background-free patterns (Nederlof *et al.*, 2013 ▸; van Genderen *et al.*, 2016 ▸).

#### 2.2.3. Structure determination   

Before data analysis, the collected images were aligned on a common center to correct for the drift of the direct beam using an in-house *MATLAB* routine. The program *PETS* (Palatinus, 2011 ▸) was used to refine the tilt-axis angle (*i.e.* the spindle direction), to determine the unit-cell vectors and to reconstruct the sections of reciprocal space used for determination of the space group.

The geometric parameters of the experiment (tilt axis, camera length, detector description *etc.*) were then converted to the conventional X-ray diffraction geometry, allowing analysis using standard programs dedicated to X-ray protein crystallography. The effective detector distance (camera length) was determined by 3D data collections from a natrolite powder for which the unit-cell parameters had previously been determined using an X-ray powder diffractometer.

*XDS* (Kabsch, 2010 ▸), which was run with default settings, confirmed the unit-cell parameters and symmetry determined by *PETS* and was used for reflection-intensity integration.

Data analysis and molecular replacement were performed using *Phaser* (McCoy *et al.*, 2007 ▸) and the *PHENIX* package (Adams *et al.*, 2010 ▸), which can include electron scattering factors. Manual model building was performed using *Coot* (Emsley *et al.*, 2010 ▸) and software included in the *CCP*4 package (Winn *et al.*, 2011 ▸). Molecular replacement was started using the coordinates of a polyalanine model (PDB entry 1b2k, monomer *A*; Vaney *et al.*, 2001 ▸) as the search structure.

For the description of the crystal packing, after excluding the solvent molecules we generated all symmetry-equivalent protein molecules with at least one atom within a distance of 4.5 Å from any atom of the asymmetric unit (Carugo & Djinović-Carugo, 2012 ▸). We used the *UCSF Chimera* package (Pettersen *et al.*, 2004 ▸) to identify the intermolecular close contacts and potential hydrogen-bond donors and acceptors in these molecules. Molecular graphics were created with *PyMOL* (v.2.2; Schrödinger) and *Mercury* v.3.10.3 (Macrae *et al.*, 2008 ▸).

### 2.3. Powder X-ray diffraction   

High-resolution X-ray powder diffraction patterns were collected on the XRD1 beamline at the Elettra synchrotron, Trieste, Italy with a beam of 200 × 200 µm in size and wavelength 1.000 Å. Data were recorded using a Dectris PILATUS 2M detector at a distance of 800 mm. The suspension of needle-shaped HEWL microcrystals was exposed to X-rays for a continuous rotation of 360° with a speed of 1.5° s^−1^ (Supplementary Fig. S2).

*FIT*2*D* was used to process the images and integrate the diffraction rings after applying masks to cover detector gaps, the beamstop and shadowed areas. The resulting pattern spanned the *d*-range 104–5 Å. A Pawley fitting was performed using *GSAS-II* (Toby & Von Dreele, 2013 ▸) using the unit-cell parameters determined by ED as a starting guess. The background was modeled with a 30th-order Chebyschev polynomial based on fixed points, while the peaks were fitted with Gaussian profiles and improved by refining uniaxial size and strain parameters. Finally, the unit-cell parameters were refined until convergence (*R*_wp_ = 1.21%, *R*_w,exp_ = 2.90%), resulting in values of *a* = 31.48 (3), *b* = 52.11 (7), *c* = 70.99 (17) Å, β = 98.91 (12)° (Supplementary Fig. S3).

### 2.4. Single-crystal microfocus X-ray diffraction   

Measurements of single-crystal X-ray diffraction were performed on the microfocus Gemini beamline ID23-2 at the European Synchrotron Radiation Facility (ESRF), Grenoble, France (Flot *et al.*, 2010 ▸) using a crystal of HEWL of just sufficient size (Supplementary Fig. S4), which grew after several weeks in the crystallization drop from which the nanocrystals for 3D ED experiments had been obtained. The measurements were carried out with a beam of 4 × 10 µm in size and wavelength 0.873 Å and were recorded using a Dectris PILATUS3 X 2M detector. The data were integrated using *XDS* and were processed using the *CCP*4 package as for the 3D ED data. Molecular replacement was performed with *Phaser* (McCoy *et al.*, 2007 ▸) using the coordinates of a polyalanine model (PDB entry 1b2k). Manual model building was performed using *Coot* (Emsley *et al.*, 2010 ▸) and software included in the *CCP*4 package (Winn *et al.*, 2011 ▸). Despite the significant multiplicity and 〈*I*/σ(*I*)〉 of the diffraction data set (Table 1 ▸), the small diffracting volume of the crystal (30  × 5 × ≤1 µm) exposed to the microfocused X-ray radiation (Supplementary Fig. S4) limited the overall data-collection completeness to ∼75% (∼79% in the highest resolution shell) and the resolution to 2.6 Å. Cycles of automatic and manual building were performed using *Coot* coupled with refinement cycles using *REFMAC*5 in *CCP*4. The final refined model included 20 chloride ions that occupied previously reported common anion sites in HEWL (Vaney *et al.*, 2001 ▸). Table 1 ▸ reports the final crystallographic analysis and refinement statistics.

## 3. Results and discussion   

### 3.1. Nanobeam 3D ED data collection   

There are two different ways of collecting data in 3D ED: the stepwise mode (STmode), in which the crystal is tilted in discrete steps and diffraction patterns are acquired sequentially, and the continuous-rotation mode (Cmode), in which diffraction patterns are continuously collected while the crystal is rotating. Hitherto, the Cmode has been the preferred option for the acquisition of ED data from proteins. It is a data-collection mode that guarantees a geometry very similar to that used for protein single-crystal X-ray experiments at the synchrotron (Arndt, 1968 ▸; Pflugrath, 1999 ▸), a contiguous integration of reciprocal space avoiding the missing wedges between stationary patterns [compare the results in Shi *et al.* (2013 ▸) with those in Nannenga *et al.* (2014 ▸)] and a very short data-collection time with reduction of the total electron dose on the sample.

**Figure 2:**
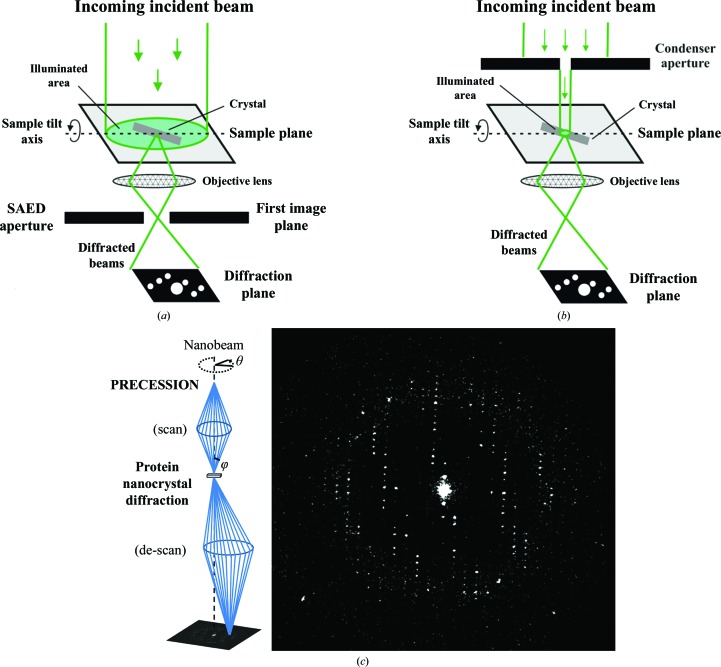
(*a*) Sketch of the illumination conditions in the case of selected-area 3D electron diffraction. (*b*) Sketch of the illumination conditions in the case of nanobeam 3D electron diffraction. (*c*) Left: beam-path geometry in precession electron diffraction. Right: single pattern collected from a nanocrystal of HEWL in precession-assisted nanobeam 3D electron diffraction mode.

However, the Cmode requires a very stable goniometer to achieve a wide reciprocal-space coverage, while in most of the currently available TEMs the stage tends to shift laterally by a few micrometres while rotating. This movement can be minimized by setting the stage at the mechanical eucentric height, but in our hands it was still a severe issue at high tilt angles when the cryo-holder tank was filled with liquid nitrogen. To compensate for such a shift, diffraction data can be collected employing large beams obtained with a post-sample selected-area ED (SAED) aperture while the sample is continuously illuminated during the 3D ED experiment (Fig. 2 ▸ *a*). In this configuration, a small lateral shift does not move the crystal completely out from the beam. There are two weaknesses to be considered: at high tilt angles the movement of the sample may be severe and unpredictable and may hamper the exploitation of the high-tilt range of the goniometer (normally ±60° in most TEMs), and the crystal is fully illuminated during the whole experiment, so it generally becomes damaged and cannot be used for a second data-acquisition trial.

An alternative beam configuration, the so-called nanobeam, can be realized with a small condenser aperture (*e.g.* 5–10 µm) in most TEMs, allowing a parallel beam of ∼30–200 nm in diameter. This configuration does not require the use of a post-sample aperture and has the advantages of a reduced background and the possibility of selectively illuminating small portions of a crystal, which may be critical in the case of crystal mosaicity.

The main advantage of the STmode is that the crystal can be re-centered after each tilt step by tracking its position by imaging. Re-centering increases the electron dose, but can allow better control of data collection and optimal exploitation of the goniometer tilt limits.

We performed the data collection in STmode, adopting and improving an experimental protocol that is largely used for the structural investigation of beam-sensitive small-molecule compounds. Preliminary crystal search and crystal tracking after each tilt step were performed in high-angle annular dark-field (HAADF) STEM imaging mode with a narrow parallel beam of ∼150 nm in diameter. The same beam conditions were also used to collect the diffraction pattern, without any further alignment. During data collection, the crystal was only illuminated in the area covered by the nanobeam (Fig. 2 ▸ *b*) and only for the exposure time necessary for diffraction data collection (2 s for lysozyme). A single STEM image required only a fraction of a second of exposure per pixel, and the related electron dose is therefore negligible. In this way, each diffraction pattern could be checked immediately after acquisition (and eventually re-acquired if not satisfactory) and a fresh undamaged area of the crystal could be selected when the original area showed hints of beam damage.

In order to properly sample the reciprocal space between the tilt steps, the beam was inclined by 0.85° and continuously precessed on a conical surface centered around the vertical axis (Fig. 2 ▸ *c*), as described by Vincent & Midgley (1994 ▸). The experimental setting was very similar to that originally described by Mugnaioli *et al.* (2009 ▸).

A low-intensity beam was used for data acquisition, corresponding to an electron dose rate of 0.01 e Å^−2^ s^−1^. Since the time spent by the beam on the crystal during the STEM imaging can be estimated to be less than 0.5 s, the total dose for the experiment (91 patterns with an exposure of 2 s each) was approximately 2 e Å^−2^. No merging of multiple data sets taken from the same or different crystals was needed to achieve structure solution.

### 3.2. Unit-cell and structure determination   

**Figure 3:**
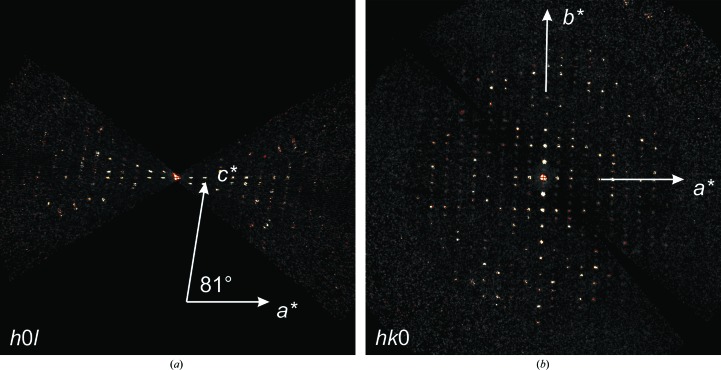
Sections of the reciprocal space of the ED data set obtained with *PETS*. (*a*) Reconstructed *h*0*l* reciprocal plane showing the monoclinic angle β* ≃ 81°. (*b*) Reconstructed *hk*0 reciprocal plane showing the reflection rule 0*k*0: *k* = 2*n*. Note that very weak intensities appear for extinct reflections owing to residual dynamic effects.

The collected ED images covered 91° of reciprocal space up to ∼2.5 Å resolution (Fig. 2 ▸ *c*). We used *PETS* (Palatinus, 2011 ▸), *XDS* (Kabsch, 2010 ▸) and the *CCP*4 software package (Winn *et al.*, 2011 ▸) to process all reflections (Section 2). Data indexing showed a monoclinic unit cell with parameters *a* = 31.9, *b* = 54.4, *c* = 71.8 Å, β = 98.8° (see Table 1 ▸). The monoclinic angle is evident from the *h*0*l* section of reciprocal space, and the condition *k* = 2*n* for the observed 0*k*0 reflections unequivocally identifies space group *P*2_1_ (Fig. 3 ▸). The determined unit cell does not correspond to any HEWL structure reported to date in the PDB. All previously solved HEWL structures belonging to space group *P*2_1_ fall into two main groups, one with unit-cell parameters *a* = 27.7, *b* = 62.8, *c* = 59.8 Å, β = 90.1° and two molecules in the asymmetric unit (for example PDB entry 1b2k; Vaney *et al.*, 2001 ▸) and the other with unit-cell parameters *a* = 26.9, *b* = 58.9, *c* = 31.3 Å, β = 110.4° and one single molecule in the asymmetric unit (for example PDB entry 1lma; Madhusudan *et al.*, 1993 ▸).

**Figure 4:**
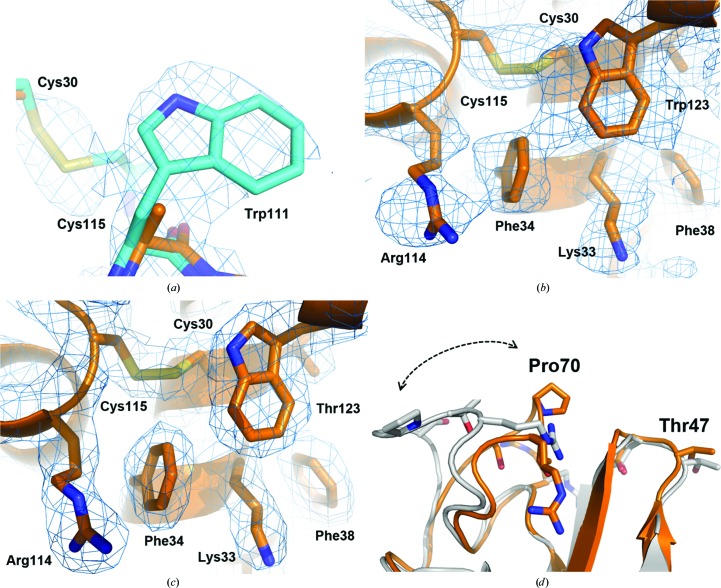
Structure of the novel HEWL polymorph obtained by 3D ED and comparison with the reported structure with PDB code 1b2k. (*a*) 2*F*_o_ − *F*_c_ map obtained by molecular replacement using the collected 3D ED data and the HEWL polyalanine model. The quality of the phasing is shown by the map (1σ) extending beyond the polyalanine model (C atoms in orange) and by the superposition of the final refined HEWL structure coordinates (C atoms in cyan). (*b*) The map (1σ) obtained by reducing the structural model bias (exclusion of the missing *F*_obs_) is locally in agreement with the side-chain orientations despite the partial distortion of the map. (*c*) Quality of the final (1σ) map obtained by filling the missing *F*_obs_ with *F*_calc_ in the region shown in (*b*). (*d*) Superposition of the refined structure coordinates (subunit *A*) with those of the monoclinic structure with PDB code 1b2k (subunit *A*) shows a relevant conformation difference in the region of the Pro70-loop (arrow).

Molecular replacement, which started with a polyalanine model (see Section 2.2.3) using *Phaser*, fitted two molecules in the asymmetric unit of the novel HEWL polymorph. The run resulted in final translation-function *Z*-score (Top TFZ) and log-likelihood gain (Top LLG) values of 14.4 and 242.5, respectively, which suggested a good molecular-replacement solution and successful experimental phasing (McCoy *et al.*, 2007 ▸). After the first refinement cycle of the polyalanine model using secondary-structure and noncrystallographic symmetry restraints, *R* and *R*_free_ were 0.41 and 0.42, respectively. The resulting OMIT map showed residual density corresponding to most of the model side chains that were missing (Fig. 4 ▸ *a*). Several rounds of model building and refinement were performed using the iterative-build OMIT map mode of *AutoBuild* (*PHENIX*) to limit model bias, and by manual map interpretation in *Coot* (Figs. 4 ▸ *b* and 4 ▸ *c*). Despite the structural restraints, the chain *B* residues Gly71 and Thr47 were converted to *cis*-peptides during refinement, possibly owing to the limited completeness of the ED data. Table 1 ▸ shows the final structure-refinement statistics.

### 3.3. Structure description   

During the process of model building and refinement, the limited completeness of the ED data collection (∼66%) caused distortion of the map, but still produced an interpretable map (Figs. 4 ▸ *b* and 4 ▸ *c*). Subunits *A* and *B* in the novel HEWL polymorph were substantially analogous (r.m.s.d. of 0.60 Å for C^α^ atomic coordinates). The refined structure showed a Pro70-loop that was closer to the Thr47-loop with respect to the conformation of the Pro70-loop in other reported structures, such as, for example, PDB entry 1b2k (Vaney *et al.*, 2001 ▸; Fig. 4 ▸ *d*).

The refined conformations of the chains of the novel monoclinic polymorph instead show a closer resemblance to the chains of the reported orthorhombic (PDB entry 4r0f; Sharma *et al.*, 2016 ▸) and tetragonal (PDB entry 5wra; Sugahara *et al.*, 2017 ▸) polymorphs. Interestingly, tetragonal crystals were often observed in the same drop as the needle-like crystals of the novel polymorph, suggesting a phase transition connected with time and crystal size.

**Figure 5:**
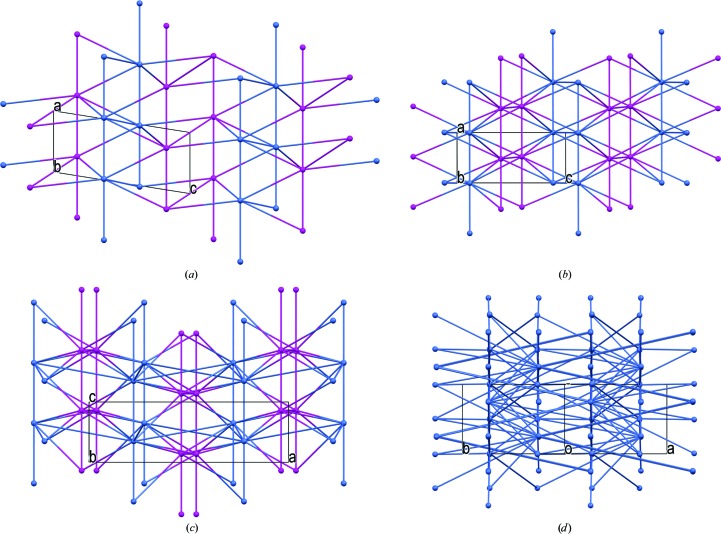
Simplified underlying nets for the examined HEWL polymorphs. Each protein chain is represented by its centroid, with different colors distinguishing symmetry-independent chains. The centroids are the nodes of the net, while the edges represent the directions in which interchain close contacts occur. (*a*) Monoclinic polymorph reported in this study, (*b*) monoclinic, PDB entry 1b2k (Vaney *et al.*, 2001 ▸); (*c*) orthorhombic, PDB entry 4r0f (Sharma *et al.*, 2016 ▸); (*d*) tetragonal, PDB entry 5wra (Sugahara *et al.*, 2017 ▸).

However, the novel monoclinic HEWL polymorph shows major differences in crystal packing from the aforementioned forms. At a first glance, the packing appears to be looser than in any known HEWL polymorph (Fig. 5 ▸). Considering only nonbridged interactions (*i.e.* protein–protein close contacts), each chain interacts with 6–8 neighboring chains, differing from the monoclinic PDB entry 1b2k (Vaney *et al.*, 2001 ▸), with 12 nearest neighbors per chain, and the orthorhombic PDB entry 4r0f (Sharma *et al.*, 2016 ▸), with 9–10 nearest neighbors per chain. Interestingly, in the tetragonal form each chain only interacts with seven neighboring chains (see Supplementary Table S1). Considering a narrower subset of intermolecular interactions, namely the potential hydrogen bonds among residues of neighboring chains, it appears that the new structure can form about half of the hydrogen bonds that are found in most known HEWL polymorphs (see Supplementary Tables S1 and S2). The new monoclinic phase may be expected to have a lower stability than the concurrently forming tetragonal phase. Nevertheless, the monoclinic crystals could be stored for several months in the crystallization drop without any apparent transformation. Finally, it appears that the residues that can form interchain hydrogen bonds are not the same in the four examined models (Supplementary Table S2); therefore, the packing of the novel monoclinic HEWL is indeed unique within the HEWL polymorphic system and cannot be traced back to a distortion of any known structure.

### 3.4. Validation by powder and single-crystal X-ray diffraction   

To validate the discovery of a novel polymorph of HEWL, we analyzed its polycrystalline ensemble by X-ray powder diffraction (Von Dreele, 2003 ▸; Margiolaki *et al.*, 2007 ▸). The HEWL crystalline phase was characterized by means of high-resolution synchrotron powder diffraction. Pawley fitting converged successfully to a monoclinic unit cell with parameters that were in agreement with the 3D ED results (Supplementary Fig. S3).

**Figure 6:**
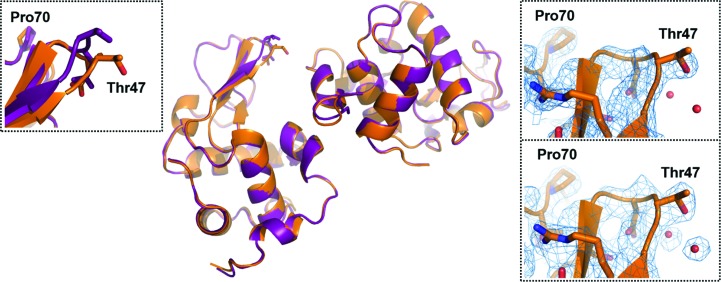
Superposition of the coordinates of the two HEWL structures obtained by 3D ED (C atoms in violet) and X-ray diffraction on the microfocus beamline (C atoms in orange). The insets on the right show the electron-density map in the region of the Thr47-loop (top panel, map calculated by exclusion of the missing *F*_obs_; bottom panel, map calculated by replacing the missing *F*_obs_ with *F*_calc_); the inset on the left shows minor structural changes of the protein localized at the Thr47-loop, where the protein seems to be more affected by the crystal-growth process.

After several weeks, we observed that some crystals in the crystallization drop from which the needle-shaped nanocrystals used for 3D ED had been obtained reached a size of a few micrometres (Fig. 1 ▸ *b* and Supplementary Fig. S4), useful for microfocus X-ray diffraction at a synchrotron facility. We succeeded in collecting an X-ray data set from one of these microcrystals (at least ∼50 times larger than the average needles in the aggregate), analysis of which showed unit-cell parameters in agreement with those obtained by 3D ED (Table 1 ▸). The X-ray crystal structure was solved and refined at ∼2.6 Å resolution using a procedure similar to that used for the ED data (Table 1 ▸). The refined model could be superimposed with that obtained by 3D ED (Fig. 6 ▸) with r.m.s.d.s of 0.36 Å for chain *A* and 0.55 Å for chain *B* (referring to C^α^ atoms), validating the crystal structure of the novel polymorph of HEWL discovered by 3D ED.

## 4. Conclusions   

The present study demonstrates the potential of 3D ED for the structural characterization of submicrometric protein crystals. In particular, this method can reveal the presence of new protein structure polymorphs that are hardly detectable by conventional X-ray diffraction. Protein nanocrystals can be relatively easy to grow (Stevenson *et al.*, 2014 ▸), require lower quantities of starting material and have potentially fewer defects (de la Cruz *et al.*, 2017 ▸) than the macroscopic crystals that are used for conventional X-ray crystallography. In contrast to XFEL experiments, which require intense femto­second pulses delivered by the world’s largest and most powerful X-ray machines and billions of nanocrystals and microcrystals (Uervirojnangkoorn *et al.*, 2015 ▸), 3D ED can provide relevant structural information from only one or a few such crystals.

The ability of HEWL to crystallize in different packing arrangements and crystal forms has been a focus of X-ray crystallography for more than fifty years. The 3D ED method allowed us to discover a novel HEWL monoclinic polymorph which is very challenging to characterize using more conventional techniques, owing to its habitus and size. Remarkably, although the data completeness was below 70%, most of the side chains could be determined by iterative manual fitting of a polyalanine starting model. This confirms that 3D ED data have sufficient quality for the discovery of new protein forms.

To collect the 3D ED data, we have applied a nanobeam precession-assisted approach to protein crystallography, which allows the illumination of only the desired area of a given submicrometric crystal and hence the acquisition of data from fresh, undamaged areas of the sample. A similar strategy is routinely applied at synchrotron microfocus beamlines in order to preserve very small plate-shaped or needle-shaped protein crystals from radiation damage (Flot *et al.*, 2010 ▸; Sanishvili *et al.*, 2011 ▸). In most cases, such a protocol maximizes the quality of the diffraction patterns over an extended angular range and allows tracking of the position of the crystal, which tends to drift in current microscope cryo-holders.

The results of our study suggest 3D ED as an approach for time-dependent monitoring of the dynamic events in protein nucleation, polymorph formation and stabilization, which can be extremely relevant for understanding biological mechanisms and has potential impacts in medicine and biomaterials science. The investigation of protein nucleation processes and the structural characterization of the first-forming polymorphs is also relevant for the use of biomolecules in industrial and pharmaceutical applications (Van Driessche *et al.*, 2018 ▸).

The success of 3D ED in entering every sector of crystallo­graphy calls for a new segment of TEMs specifically designed and dedicated to diffraction (Gemmi *et al.*, 2015 ▸). The stability of the sample holder during tilt and the possibility of low-dose diffraction procedures are the key points that should be addressed for 3D ED to become a routine technique on very beam-sensitive samples such as biological macromolecules and organics in general. The availability of large amounts of ED data on small protein crystals is likely to push forward the search for dedicated phasing methods, with the aim of overcoming the limits of molecular replacement (Yonekura *et al.*, 2015 ▸; Ma *et al.*, 2017 ▸).

## Supplementary Material

PDB reference: hen egg-white lysozyme, X-ray microfocus diffraction structure, 6ht2

PDB reference: electron diffraction structure, 6hu5

Supplementary Figures and Tables.. DOI: 10.1107/S2052252518017657/eh5001sup1.pdf

## Figures and Tables

**Table 1:** Summary of the diffraction data and refinement statistics Values in parentheses are for the highest resolution shell.

	3D ED	X-ray diffraction
Wavelength (Å)	0.0335	0.8731
Space group	*P*2_1_	*P*2_1_
*a*, *b*, *c* (Å)	31.85, 54.38, 71.79	31.71, 53.50, 71.60
α, β, γ (°)	90.00, 98.82, 90.00	90.0, 99.10, 90.0
No. of crystals	1	1
Crystal size (µm)	∼1 × 1 × ≤0.1	∼30 × 5 × ≤1
Molecules per asymmetric unit	2	2
Resolution (Å)	42.67–2.80 (2.97–2.80)	70.72–2.60 (2.72–2.60)
Total No. of observations	10822	19412
No. of unique reflections	3906	5529
*R*_merge_	0.49 (0.88)	0.24 (0.84)
〈*I*/σ(*I*)〉	1.6 (0.6)	5.3 (2.7)
Multiplicity	2.8 (2.7)	3.5 (3.4)
Completeness (%)	66.1 (66.7)	74.6 (79.1)
*R*_work_/*R*_free_	0.29/0.34	0.20/0.25
R.m.s.d., bonds (Å)	0.01	0.01
R.m.s.d., angles (°)	0.78	1.50
Protein atom *B* factor (Å)	10.9	18.5
Ramachandran favored (%)	93.3	93.7
Ramachandran allowed (%)	4.0	4.7
