# Supplementary material for: Nanobeam precession-assisted 3D electron diffraction reveals a new polymorph of hen egg-white lysozyme
Source: IUCrJ. 2019 Jan 15;6(Pt 2):178–88. doi: 10.1107/S2052252518017657 (PMC6400191; doi:10.1107/S2052252518017657)
Supplement: Supplementary file 1 [file m-06-00178-sup1.pdf]

IUCrJ

Volume 6 (2019)

Supporting information for article:

Nanobeam precession-assisted 3D electron diffraction reveals a new polymorph of hen egg-white lysozyme

Arianna Lanza, Eleonora Margheritis, Enrico Mugnaioli, Valentina Cappello, Gianpiero Garau and Mauro Gemmi

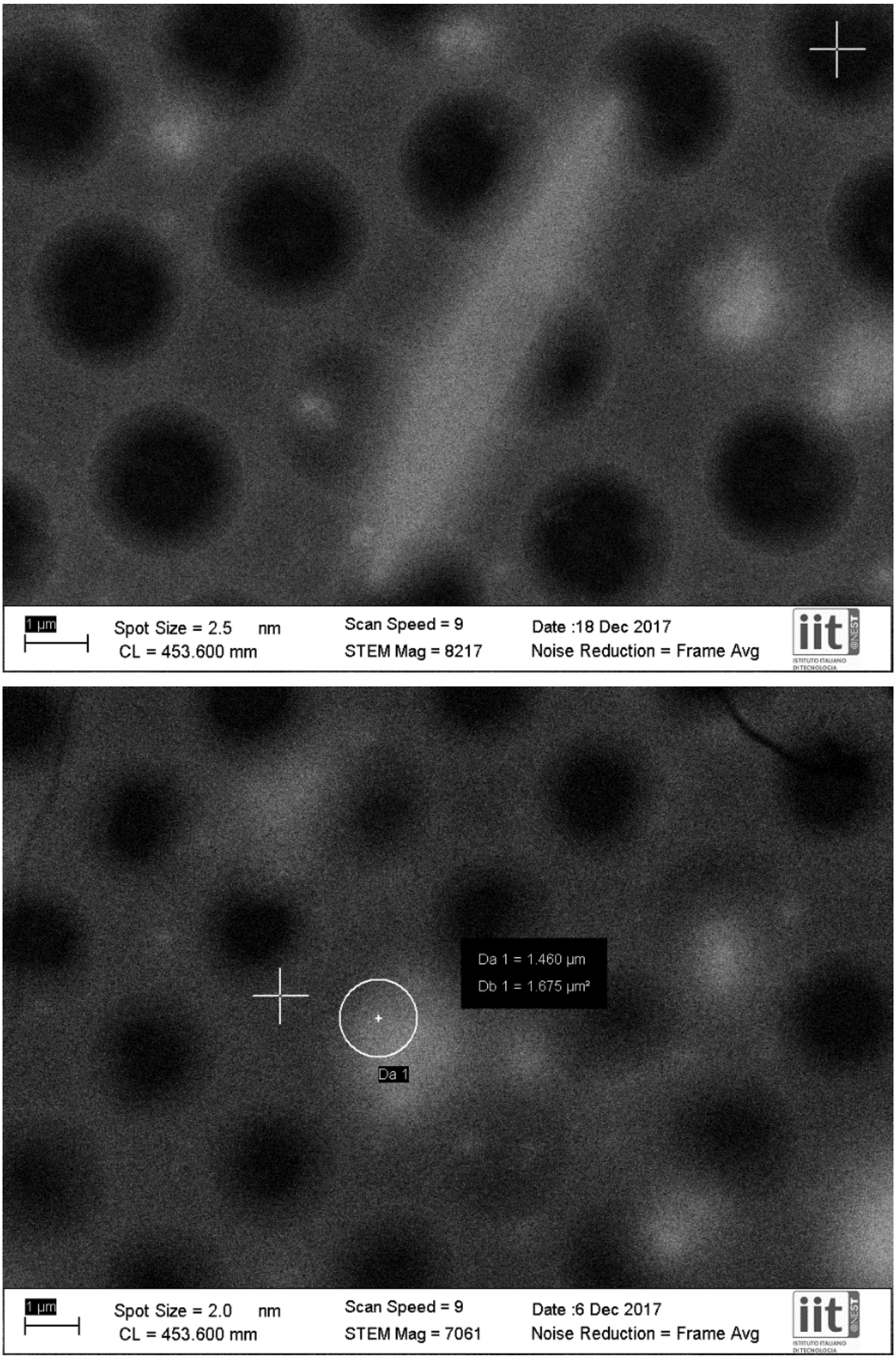

Figure S1 STEM images of plunge-frozen HEWL samples.Tipically some crystals preserving the acicular habitus can be found (top image), but in this study the best diffraction was obtained by one fragment of ca. 1µm × 1µm × ≤ 0.1µm, like the one in the bottom image. (Note: the white circle is not related with the size of the beam, it was only used to highlight the diffracting area exploited during the whole data collection.)

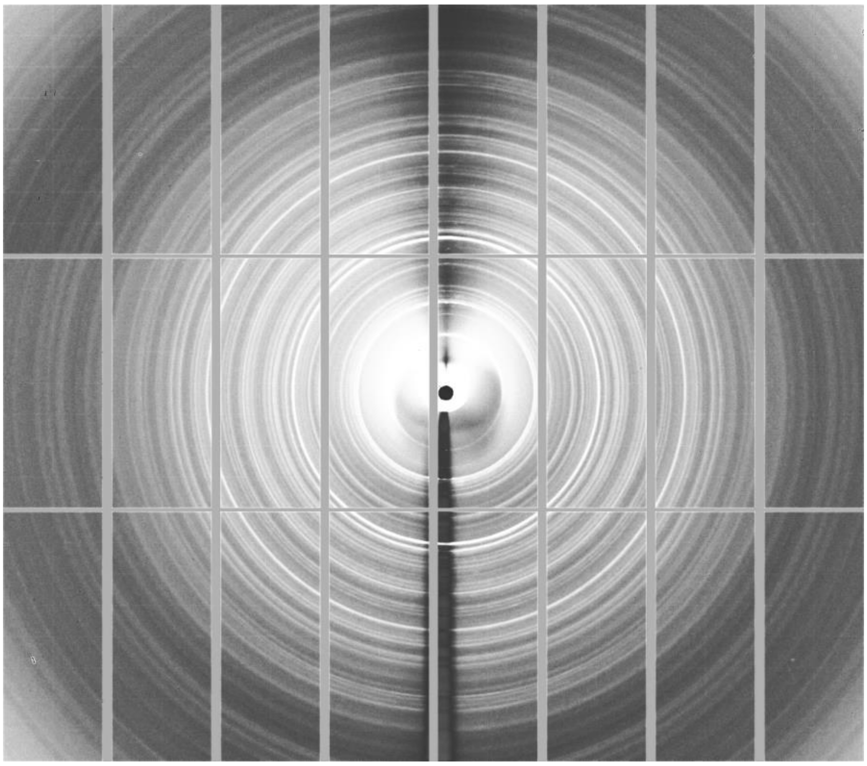

Figure S2 High resolution powder diffraction image of the HEWL aggregate, collected at the XRD1 Beamline of ELETTRA synchrotron, with λ=1 Å.

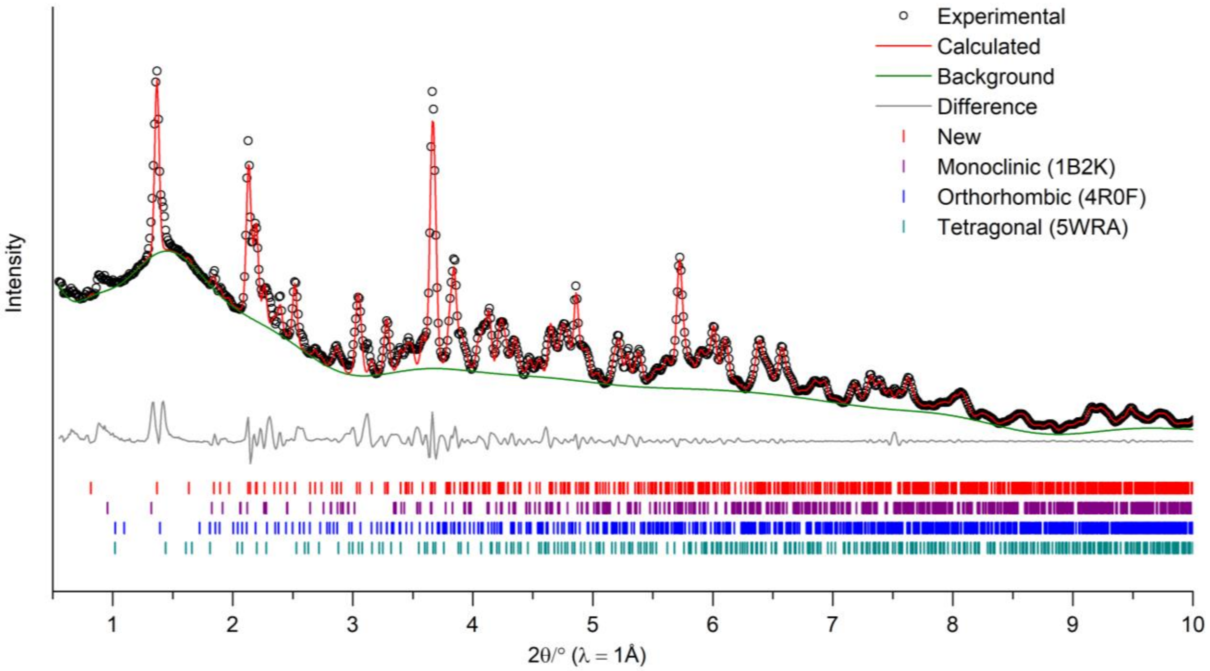

Figure S3 X-ray powder diffraction pattern obtained from the HEWL polycrystalline ensemble (black points), and the corresponding Pawley fit for the new $P2_1$ cell parameters of HEWL (red, PDB 6HT2). For comparison, the peak positions of several known phases (see the PDB entries indicated in the legend) are shown.

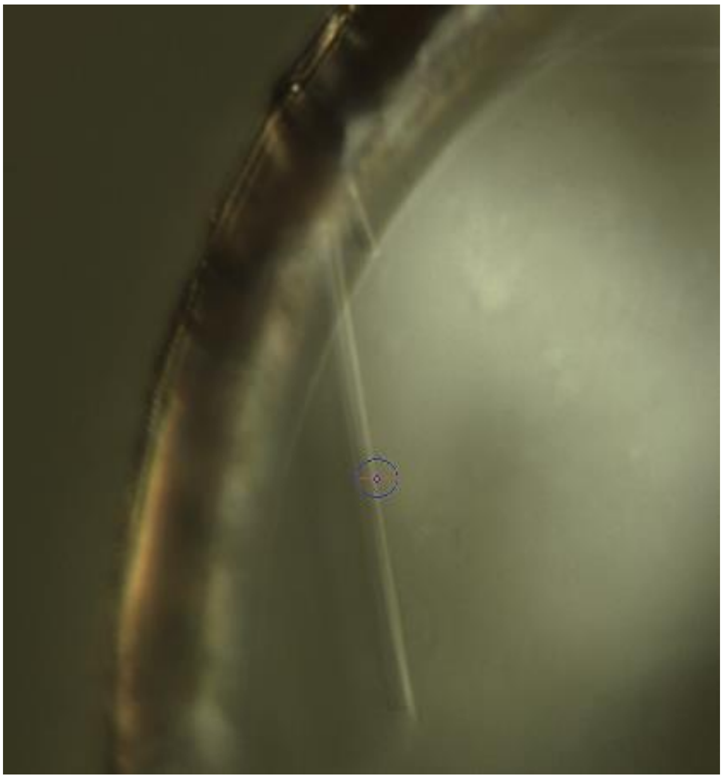

Figure S4 Single crystal used for the microfocus XRD experiment, mounted on a loop. The blue circle is ca. 4 µm in diameter.

Table S1 Summary of the potential interchain H-bonds for monoclinic, orthorhombic and tetragonal HEWL polymorphs, calculated with the program UCSF Chimera.

	mono<br>6HT2	mono<br>1B2K	ortho<br>4R0F	tetra<br>5WRA
interacting chains	8/6	12/12	10/9	8
possible H-bonds in the asymmetric unit	23	42	46	26
# per chain	11.5	21	23	26

Table S2 List of the potential interchain H-bonds for monoclinic, orthorhombic and tetragonal HEWL polymorphs, calculated with the program UCSF Chimera.

MONOCLINIC 6HT2

Donor				Acceptor				
sym id	Residue type	Residue n. / chain	D atom	sym id	Residue type	Residue n. / chain	A atom	D···A /Å
#0	CYS	6.A	SG	#9	CYS	127.B	O	3.6
#0	ARG	21.A	NH1	#2	ASP	66.A	O	2.9
#0	ARG	21.A	NH2	#2	ASP	66.A	O	3.5
#0	ARG	68.A	NH1	#7	ARG	21.A	O	2.7
#0	ARG	68.A	NH2	#7	ARG	21.A	O	3.2
#0	ARG	73.A	NH1	#6	ARG	68.B	O	3.4
#0	ARG	73.A	NH1	#8	GLY	102.B	O	3.3
#0	ARG	112.A	NH2	#3	ASN	77.A	OD1	3.2
#0	ASN	113.A	ND2	#3	ASN	77.A	OD1	3.5
#0	ASN	19.B	ND2	#7	SER	81.B	O	2.7
#0	ARG	21.B	NH1	#7	ASP	66.B	O	2.9
#0	ARG	21.B	NH2	#7	ASP	66.B	O	3.5
#0	ARG	68.B	NH1	#2	ARG	21.B	O	3.2
#0	ARG	68.B	NH2	#2	ARG	21.B	O	3.1
#0	ARG	73.B	NH1	#3	ARG	68.A	O	2.7
#0	ASN	113.B	ND2	#0	ALA	122.A	O	2.7
#8	ARG	73.B	NH1	#0	ARG	68.A	O	2.7
#2	ARG	68.A	NH1	#0	ARG	21.A	O	2.7
#2	ARG	68.A	NH2	#0	ARG	21.A	O	3.2
#2	ASN	19.B	ND2	#0	SER	81.B	O	2.7
#2	ARG	21.B	NH1	#0	ASP	66.B	O	2.9
#2	ARG	21.B	NH2	#0	ASP	66.B	O	3.5
#3	ARG	73.A	NH1	#0	GLY	102.B	O	3.3
#4	ARG	73.A	NH1	#0	ARG	68.B	O	3.4
#5	CYS	6.A	SG	#0	CYS	127.B	O	3.6
#7	ARG	21.A	NH1	#0	ASP	66.A	O	2.9
#7	ARG	21.A	NH2	#0	ASP	66.A	O	3.5
#7	ARG	68.B	NH1	#0	ARG	21.B	O	3.2
#7	ARG	68.B	NH2	#0	ARG	21.B	O	3.1
#8	ARG	112.A	NH2	#0	ASN	77.A	OD1	3.2
#8	ASN	113.A	ND2	#0	ASN	77.A	OD1	3.5

TETRAGONAL 5WRA

Donor				Acceptor				
sym id	Residue type	Residue n. / chain	D atom	sym id	Residue type	Residue n. / chain	A atom	D···A /Å
#0	LYS	13.A	NZ	#5	LEU	129.A	O	2.8
#0	ASN	39.A	ND2	#3	ASP	66.A	O	2.9
#0	GLN	41.A	NE2	#3	ASN	65.A	O	3.1
#0	THR	47.A	OG1	#8	GLY	126.A	O	2.7
#0	ARG	68.A	NH1	#3	THR	43.A	O	2.7
#0	ARG	68.A	NH2	#3	THR	43.A	O	3.1
#0	SER	81.A	N	#3	GLN	41.A	OE1	2.8
#0	SER	81.A	OG	#3	GLN	41.A	OE1	3.6
#0	ASN	106.A	ND2	#4	ASN	113.A	O	3.0
#0	ARG	112.A	NH2	#6	GLY	102.A	O	3.1
#0	ASN	113.A	ND2	#6	GLY	102.A	O	3.0
#0	ARG	114.A	NH1	#6	GLY	22.A	O	2.9
#0	LYS	116.A	NZ	#4	ASN	113.A	O	3.0
#0	ARG	128.A	NE	#2	THR	47.A	O	3.2
#0	ARG	128.A	NH1	#5	ARG	14.A	O	3.2
#2	THR	47.A	OG1	#0	GLY	126.A	O	2.7
#3	ASN	39.A	ND2	#0	ASP	66.A	O	2.9
#3	GLN	41.A	NE2	#0	ASN	65.A	O	3.1
#3	ARG	68.A	NH1	#0	THR	43.A	O	2.7
#3	ARG	68.A	NH2	#0	THR	43.A	O	3.1
#3	SER	81.A	N	#0	GLN	41.A	OE1	2.8
#3	SER	81.A	OG	#0	GLN	41.A	OE1	3.6
#4	ARG	112.A	NH2	#0	GLY	102.A	O	3.1
#4	ASN	113.A	ND2	#0	GLY	102.A	O	3.0
#4	ARG	114.A	NH1	#0	GLY	22.A	O	2.9
#5	LYS	13.A	NZ	#0	LEU	129.A	O	2.8
#5	ARG	128.A	NH1	#0	ARG	14.A	O	3.2
#6	ASN	106.A	ND2	#0	ASN	113.A	O	3.0
#6	LYS	116.A	NZ	#0	ASN	113.A	O	3.0
#8	ARG	128.A	NE	#0	THR	47.A	O	3.2

Donor				Acceptor					Donor				Acceptor				
sym id	Residue type	Residue n. / chain	D atom	sym id	Residue type	Residue n. / chain	A atom	D··A /Å	sym id	Residue type	Residue n. / chain	D atom	sym id	Residue type	Residue n. / chain	A atom	D··A /Å
ORTHORHOMBIC 4R0F									MONOCLINIC 1B2K								
#0	ARG	14.A	NH1	#0	ASN	37.B	O	3.1	#0	ARG	5.A	NH2	#13	ASP	101.A	O	2.9
#0	ASN	19.A	ND2	#4	SER	81.A	O	2.8	#0	ASN	39.A	ND2	#7	GLN	121.B	OE1	3.1
#0	ASN	19.A	ND2	#4	GLY	126.B	O	3.1	#0	ARG	73.A	NH2	#4	ASP	119.A	OD2	2.9
#0	ARG	61.A	NH1	#12	GLY	67.B	O	3.1	#0	LYS	97.A	NZ	#2	ARG	45.A	O	3.2
#0	ARG	61.A	NH2	#10	ASP	101.B	O	2.5	#0	ARG	112.A	NH1	#4	ARG	128.A	O	3.3
#0	ARG	68.A	NH1	#11	ARG	21.A	O	2.8	#0	ARG	112.A	NH2	#4	CYS	127.A	O	2.7
#0	ARG	68.A	NH2	#11	ARG	21.A	O	3.0	#0	ARG	112.A	NH2	#4	ARG	128.A	O	3.4
#0	ARG	73.A	NH2	#12	ARG	68.B	O	2.5	#0	ARG	114.A	NH1	#7	ASP	18.A	O	3.6
#0	ASN	77.A	ND2	#3	ASP	119.A	OD2	2.8	#0	ARG	125.A	NE	#13	ASP	101.A	OD2	2.9
#0	ASN	103.A	N	#12	GLY	71.B	O	2.8	#0	ARG	125.A	NH2	#13	ASP	101.A	OD1	3.1
#0	ARG	114.A	NE	#13	SER	86.B	OG	3.1	#0	ARG	128.A	NH2	#13	ASP	52.A	OD1	3.4
#0	GLN	121.A	NE2	#13	ASN	77.A	OD1	3.0	#0	LYS	1.B	NZ	#6	GLY	71.A	O	3.5
#0	ARG	125.A	NH2	#13	ARG	73.A	O	3.5	#0	VAL	2.B	N	#12	ASP	48.A	O	3.2
#0	ARG	125.A	NH2	#13	ASN	74.A	O	3.0	#0	ARG	5.B	N	#6	GLY	102.B	O	3.1
#0	ARG	128.A	N	#9	THR	47.B	O	2.9	#0	ARG	5.B	NE	#6	ASP	101.B	O	3.0
#0	ARG	128.A	NH1	#9	GLY	49.B	O	3.5	#0	ARG	5.B	NH2	#6	ASP	101.B	O	3.0
#0	ARG	128.A	NH2	#9	ASN	46.B	O	2.9	#0	CYS	6.B	N	#6	ASN	103.B	OD1	2.7
#0	ARG	128.A	NH2	#9	SER	50.B	O	2.9	#0	ARG	14.B	NH1	#6	PRO	70.A	O	2.6
#0	ARG	5.B	N	#0	ASP	87.A	OD2	3.3	#0	ARG	14.B	NH2	#6	PRO	70.A	O	3.0
#0	ASN	19.B	ND2	#11	SER	81.B	O	2.7	#0	ASN	19.B	ND2	#2	LEU	84.A	O	3.3
#0	ARG	68.B	NH1	#4	ARG	21.B	O	3.1	#0	ARG	68.B	NH1	#10	GLY	22.A	O	2.9
#0	ARG	68.B	NH2	#4	ARG	21.B	O	3.1	#0	SER	81.B	OG	#9	ASN	113.A	O	2.7
#0	ARG	73.B	NH1	#2	GLY	49.A	O	2.8	#0	ARG	114.B	NH1	#7	ARG	14.B	O	3.1
#0	ARG	73.B	NH1	#2	ARG	68.A	O	2.9	#0	LYS	116.B	NZ	#0	ASN	77.A	OD1	3.4
#0	SER	85.B	OG	#3	ASN	113.A	O	2.7	#0	ASP	119.B	N	#0	ASP	87.A	OD2	2.8
#0	THR	89.B	OG1	#3	LYS	116.A	O	2.8	#0	GLN	121.B	NE2	#2	ASN	39.A	OD1	3.6
#2	ARG	61.A	NH2	#0	ASP	101.B	O	2.5	#2	ASN	39.A	ND2	#0	GLN	121.B	OE1	3.1
#3	ARG	114.A	NE	#0	SER	86.B	OG	3.1	#2	ARG	114.A	NH1	#0	ASP	18.A	O	3.6
#3	GLN	121.A	NE2	#0	ASN	77.A	OD1	3.0	#2	ARG	114.B	NH1	#0	ARG	14.B	O	3.1
#3	ARG	125.A	NH2	#0	ARG	73.A	O	3.5	#3	VAL	2.B	N	#0	ASP	48.A	O	3.2
#3	ARG	125.A	NH2	#0	ASN	74.A	O	3.0	#4	ARG	5.A	NH2	#0	ASP	101.A	O	2.9
#4	ARG	68.A	NH1	#0	ARG	21.A	O	2.8	#4	ARG	125.A	NE	#0	ASP	101.A	OD2	2.9
#4	ARG	68.A	NH2	#0	ARG	21.A	O	3.0	#4	ARG	125.A	NH2	#0	ASP	101.A	OD1	3.1
#4	ASN	19.B	ND2	#0	SER	81.B	O	2.7	#4	ARG	128.A	NH2	#0	ASP	52.A	OD1	3.4
#5	ARG	61.A	NH1	#0	GLY	67.B	O	3.1	#5	LYS	1.B	NZ	#0	GLY	71.A	O	3.5
#5	ARG	73.A	NH2	#0	ARG	68.B	O	2.5	#5	ARG	5.B	N	#0	GLY	102.B	O	3.1
#5	ASN	103.A	N	#0	GLY	71.B	O	2.8	#5	ARG	5.B	NE	#0	ASP	101.B	O	3.0
#9	ARG	128.A	N	#0	THR	47.B	O	2.9	#5	ARG	5.B	NH2	#0	ASP	101.B	O	3.0
#9	ARG	128.A	NH1	#0	GLY	49.B	O	3.5	#5	CYS	6.B	N	#0	ASN	103.B	OD1	2.7
#9	ARG	128.A	NH2	#0	ASN	46.B	O	2.9	#5	ARG	14.B	NH1	#0	PRO	70.A	O	2.6
#9	ARG	128.A	NH2	#0	SER	50.B	O	2.9	#5	ARG	14.B	NH2	#0	PRO	70.A	O	3.0
#10	ARG	73.B	NH1	#0	GLY	49.A	O	2.8	#7	LYS	97.A	NZ	#0	ARG	45.A	O	3.2
#10	ARG	73.B	NH1	#0	ARG	68.A	O	2.9	#7	ASN	19.B	ND2	#0	LEU	84.A	O	3.3

#11	ASN	19.A	ND2	#0	SER	81.A	O	2.8
#11	ASN	19.A	ND2	#0	GLY	126.B	O	3.1
#11	ARG	68.B	NH1	#0	ARG	21.B	O	3.1
#11	ARG	68.B	NH2	#0	ARG	21.B	O	3.1
#13	ASN	77.A	ND2	#0	ASP	119.A	OD2	2.8
#13	SER	85.B	OG	#0	ASN	113.A	O	2.7
#13	THR	89.B	OG1	#0	LYS	116.A	O	2.8

#7	GLN	121.B	NE2	#0	ASN	39.A	OD1	3.6
#8	SER	81.B	OG	#0	ASN	113.A	O	2.7
#11	ARG	68.B	NH1	#0	GLY	22.A	O	2.9
#13	ARG	73.A	NH2	#0	ASP	119.A	OD2	2.9
#13	ARG	112.A	NH1	#0	ARG	128.A	O	3.3
#13	ARG	112.A	NH2	#0	CYS	127.A	O	2.7
#13	ARG	112.A	NH2	#0	ARG	128.A	O	3.4
